# GPR88 Reveals a Discrete Function of Primary Cilia as Selective Insulators of GPCR Cross-Talk

**DOI:** 10.1371/journal.pone.0070857

**Published:** 2013-08-02

**Authors:** Aaron Marley, Regina Wai-Yan Choy, Mark von Zastrow

**Affiliations:** Departments of Psychiatry and Cellular and Molecular Pharmacology, University of California San Francisco, San Francisco, California, United States of America; Monell Chemical Senses Center, United States of America

## Abstract

A number of G protein-coupled receptors (GPCRs) localize to primary cilia but the functional significance of cilia to GPCR signaling remains incompletely understood. We investigated this question by focusing on the D1 dopamine receptor (D1R) and beta-2 adrenergic receptor (B2AR), closely related catecholamine receptors that signal by stimulating production of the diffusible second messenger cyclic AMP (cAMP) but differ in localization relative to cilia. D1Rs robustly concentrate on cilia of IMCD3 cells, as shown previously in other ciliated cell types, but disrupting cilia did not affect D1R surface expression or ability to mediate a concentration-dependent cAMP response. By developing a FRET-based biosensor suitable for resolving intra- from extra- ciliary cAMP changes, we found that the D1R-mediated cAMP response is not restricted to cilia and extends into the extra-ciliary cytoplasm. Conversely the B2AR, which we show here is effectively excluded from cilia, also generated a cAMP response in both ciliary and extra-ciliary compartments. We identified a distinct signaling effect of primary cilia through investigating GPR88, an orphan GPCR that is co-expressed with the D1R in brain, and which we show here is targeted to cilia similarly to the D1R. In ciliated cells, mutational activation of GPR88 strongly reduced the D1R-mediated cAMP response but did not affect the B2AR-mediated response. In marked contrast, in non-ciliated cells, GPR88 was distributed throughout the plasma membrane and inhibited the B2AR response. These results identify a discrete ‘insulating’ function of primary cilia in conferring selectivity on integrated catecholamine signaling through lateral segregation of receptors, and suggest a cellular activity of GPR88 that might underlie its effects on dopamine-dependent behaviors.

## Introduction

Primary cilia are complex plasma membrane-associated molecular machines that play important roles in cellular signal transduction. Cilia are well known to facilitate sensory signaling by positioning light and odorant -activated G protein-coupled receptors (GPCRs) in close physical proximity to their cognate sensory stimuli [1] and, in vertebrate cells, are required for generating appropriately graded signaling responses to locally deposited hedgehog morphogens. A number of GPCRs that are activated by freely diffusible ligands also localize to cilia but, for such ‘conventional’ GPCRs, the functional significance of cilia is less clear [2], [3], [4], [5], [6], [7]. There is evidence that primary cilia function to localize these signals as well, such as by organizing phosphodiesterases that limit spread of the downstream signal [8] and promoting GPCR oligomer formation in the cilium [9]. Might primary cilia afford additional functional advantages to GPCR signaling elicited by diffusible ligands?

We investigated this question in a simple cell culture model, focusing on the D1 dopaminergic receptor (D1R) and beta-2 adrenergic receptor (B2AR) that represent closely related catecholamine-activated GPCRs, and which mediate downstream signal transduction by stimulating cytoplasmic accumulation of the diffusible second messenger cyclic AMP (cAMP). We show that D1Rs are concentrated on the surface of primary cilia in this model and that B2ARs are largely excluded from cilia, and thus have an essentially reciprocal surface distribution. We did not observe a major effect of primary cilia in facilitating or localizing the receptor-mediated cAMP response. In the process of exploring the orphan GPCR GPR88 that is endogenously co-expressed with D1Rs in brain [10], [11], however, we uncovered evidence for a discrete function of primary cilia in enhancing the selectivity of integrated catecholamine signaling by restricting receptor cross-regulation.

## Results

### Primary cilia are not essential for graded D1R-mediated signaling

**Figure 1:**
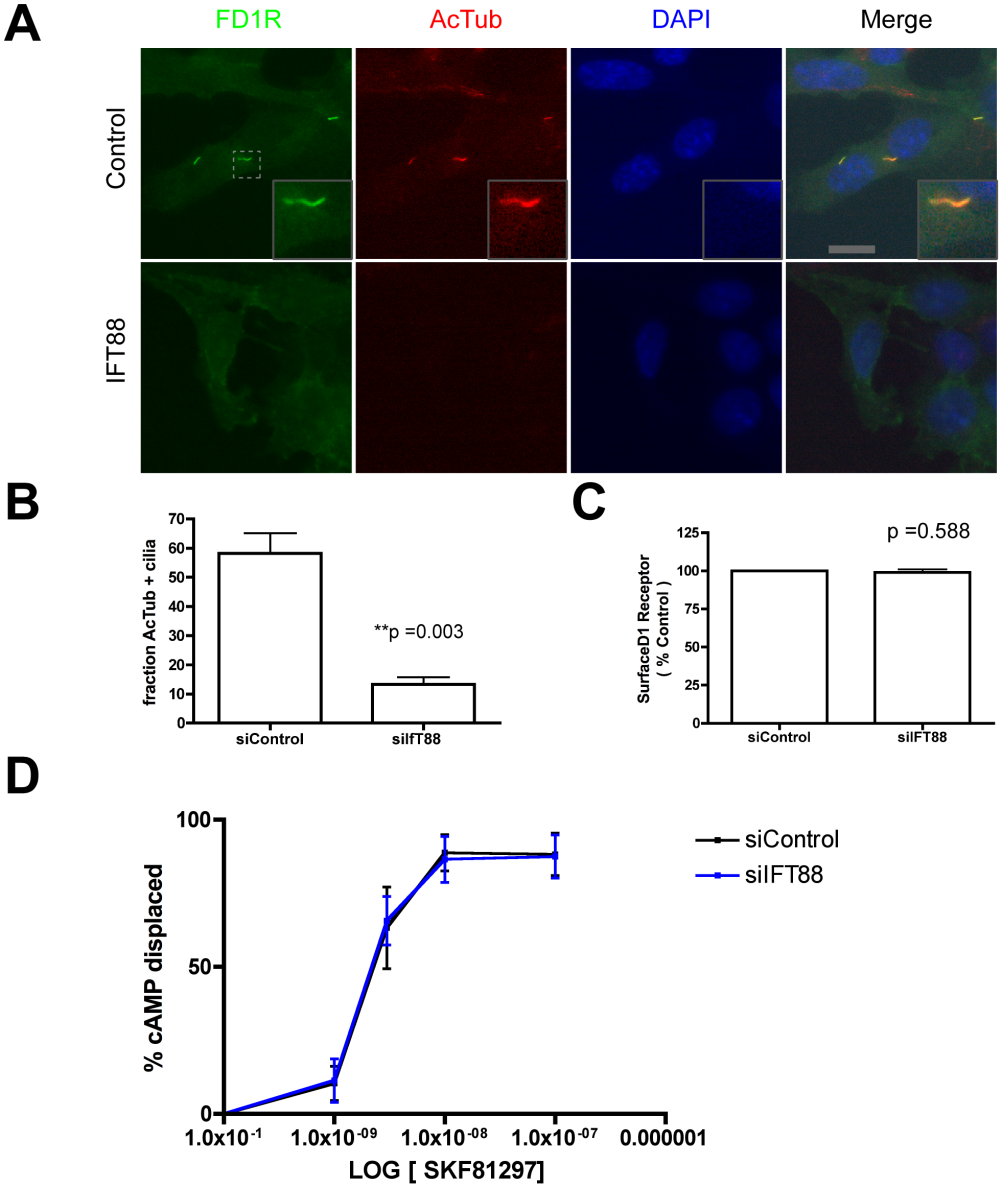
Disrupting primary cilia does not attenuate the D1R-mediated cAMP response. **A**. Representative fluorescence micrographs showing localization of surface-labeled Flag-D1 dopamine receptors (‘FD1R’, green) relative to acetylated tubulin (‘AcTub’, red) and DAPI (blue), in IMCD3 cells transfected with non-silencing RNA duplex (‘Control’, top row) or siRNA targeting IFT88 (‘IFT88’, bottom row). In control cells FD1R was concentrated in cilia marked by AcTub and also localized in the plasma membrane outside cilia (overlay shown in Merge, with an example in inset). In IFT88 knockdown cells, FD1R was diffusely localized in the plasma membrane and cilia were not detectable by either FD1R or AcTub localization. Scale bar, 10 um. Inset is displayed at 3× increased magnification. **B**. Quantification of the percentage of cells, marked by DAPI, displaying a primary cilium, marked by AcTub, in control (left bar) and IFT88 knockdown (right bar) specimens (n = 3 experiments, 200 cells counted per experiment; p value as indicated). **C**. Flow cytometric quantification of surface FD1R immunoreactivity in control relative to IFT88 knockdown cells (n = 3 experiments, each condition determined in triplicate, 10,000 cells per determination; p value as indicated). **D**. Concentration-response relationship for FD1R-mediated accumulation of cAMP measured in cell lysates using enzyme-linked immunosorbent assay.

Because D1Rs signal primarily by G protein (Gs and Golf)-linked activation of adenylyl cyclase and accumulation of the soluble cytoplasmic mediator cAMP, we first asked if primary cilia are essential for supporting the concentration-dependent cAMP response at the whole-cell level. We did so using kidney collecting duct-derived IMCD3 cells because, in our hands, IMCD3 cells do not express endogenous D1R activity as indicated by a lack of cAMP response to a saturating concentration (1 uM) of the D1-specific agonist SKF81297 (not shown). We verified that the majority (∼60%) of IMCD3 cells were ciliated under our culture conditions, and verified efficient targeting of a Flag-tagged version of the human D1R to primary cilia in these cells (**Fig. 1A**) as shown previously in other cell types. Also as expected [12], depleting IFT88 by RNA interference greatly reduced the fraction of cells expressing a primary cilium marked by acetylated tubulin immunoreactivity (**Fig. 1B**). In non-ciliated cells, D1Rs were still present in the plasma membrane but localized diffusely (Fig. 1A). Quantification of surface receptor immunoreactivity by fluorescence flow cytometry indicated that IFT88 depletion did not significantly alter overall D1R surface expression (**Fig. 1C**). To ask if cilia are essential for a graded D1R-mediated cAMP response, we measured cAMP accumulation in whole cell extracts prepared after exposing cells to the D1R-specific agonist SKF81297 for 15 min. We then compared the cAMP response observed in control cells to that observed in cells transfected with IFT88 siRNA. Control and IFT88 knockdown cells exhibited indistinguishable concentration-effect plots (**Fig. 1D**), indicating that primary cilia are not essential to produce a graded D1R-mediated signaling response.

### Primary cilia do not impose a significant barrier to diffusion of the cAMP signal

**Figure 2:**
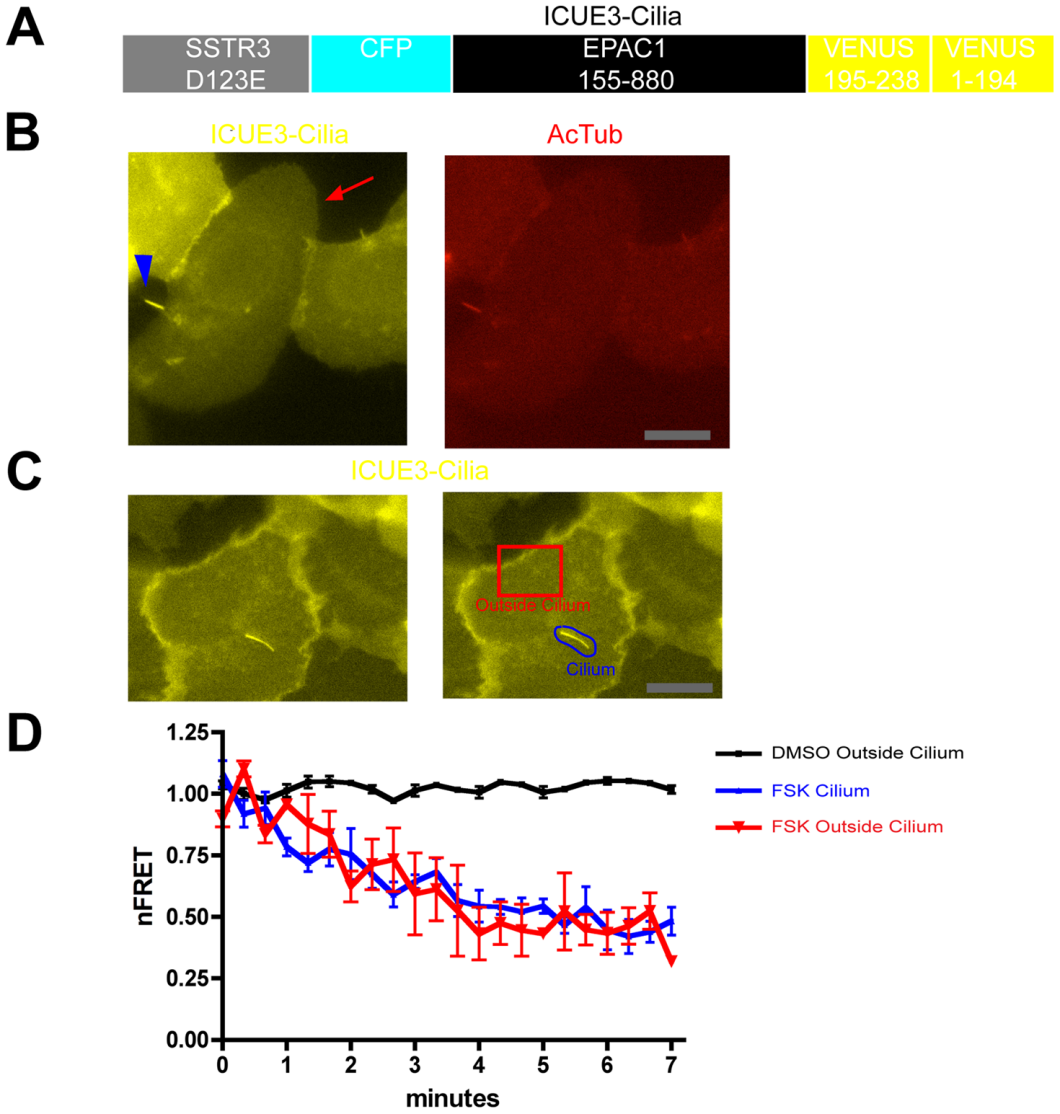
A FRET-based biosensor for local analysis of cAMP dynamics in individual cells. **A**. Schematic representation of ICUE3-Cilia design. The biosensor consists of a ligand binding-defective SSTR3 to achieve ciliary localization fused to ICUE2 except with the C-terminal citrine present in ICUE2 replaced by a circularly permuted YFP variant to increase the overall fluorescence signal. **B**. Representative fluorescence micrograph of IMCD3 cells transfected with ICUE3-Cilia, showing YFP fluorescence (left panel, yellow) relative to AcTub immunoreactivity (right panel, red). ICUE3-Cilia localized both to the primary cilium (arrowhead) and was detectable in the extra-ciliary plasma membrane (arrow). Scale bar, 10 um. **C**. YFP image of a representative ICUE3-Cilia -transfected cell (left panel), with selected regions of plasma membrane outside of cilium (‘cell’, red outline) and including the primary cilium (‘cilia’, blue outline) indicated. nFRET determinations in each region were carried out as described in Experimental Procedures. Scale bar, 10 um. **D**. Time course of nFRET change in the indicated regions of IMCD3 cells expressing ICUE3-Cilia, with 5 uM forskolin or vehicle (DMSO) applied at t = 40 sec. The black line shows vehicle control indicating minimal photobleaching over the period of nFRET determination. The red line indicates extra-ciliary (‘outside cilium’) nFRET and blue line indicates ciliary (‘cilium’) nFRET calculated from the same image series (n = 3 experiments, ≥3 cells imaged per experiment, error bars indicate S.E.M. across experiments).

We next investigated the subcellular localization of the cAMP response elicited by D1R activation in ciliated cells. FRET-based cAMP biosensors have proven very useful for assessing subcellular cAMP dynamics, as reviewed elsewhere [13], [14], but we were unable to identify an existing biosensor construct that achieved sufficient ciliary expression to allow reliable detection of cAMP accumulation in this compartment. Thus we developed a cilia targeted biosensor for this purpose. We started with a previously characterized Epac1-based biosensor, ICUE2 [15], and sought to enhance its localization to cilia. A number of modular ciliary localization signals identified previously were tried without success (not shown), but found that fusing ICUE2 to the full-length somatostatin 3 (SSTR3) promoted significant localization of the biosensor to cilia. To avoid potential complications of ligand-induced signaling by the fused SSTR3, we introduced a point mutation into the fused receptor (corresponding to the D123E mutation) that prevents ligand binding [16]. Third, to enhance the dynamic range of FRET change, we replaced the citrine present in ICUE2 with circularly permuted Venus [17], essentially mimicking the design of ICUE3 described previously by the Zhang lab [18]. The modified cAMP biosensor, which we accordingly called ‘ICUE3-Cilia’ (**Fig. 2A**), localized both to the cilium marked by acetylated tubulin and was also was distributed diffusely in the surrounding extra-ciliary plasma membrane (**Fig. 2B**), making it favorable for detecting cAMP changes in both ciliary and extra-ciliary cytoplasmic compartments. To test the ability of ICUE3-Cilia to detect cAMP in both locations, we carried out spatially resolved FRET imaging (**Fig. 2C**). Forskolin (5 uM), a general activator of adenylyl cyclase activity, produced a similarly rapid and robust reduction of normalized FRET (nFRET) measured from ICUE3-Cilia localized both to the cilium and to the extra-ciliary plasma membrane (**Fig. 2D**), verifying the ability of the biosensor to detect cAMP accumulation in both compartments.

**Figure 3:**
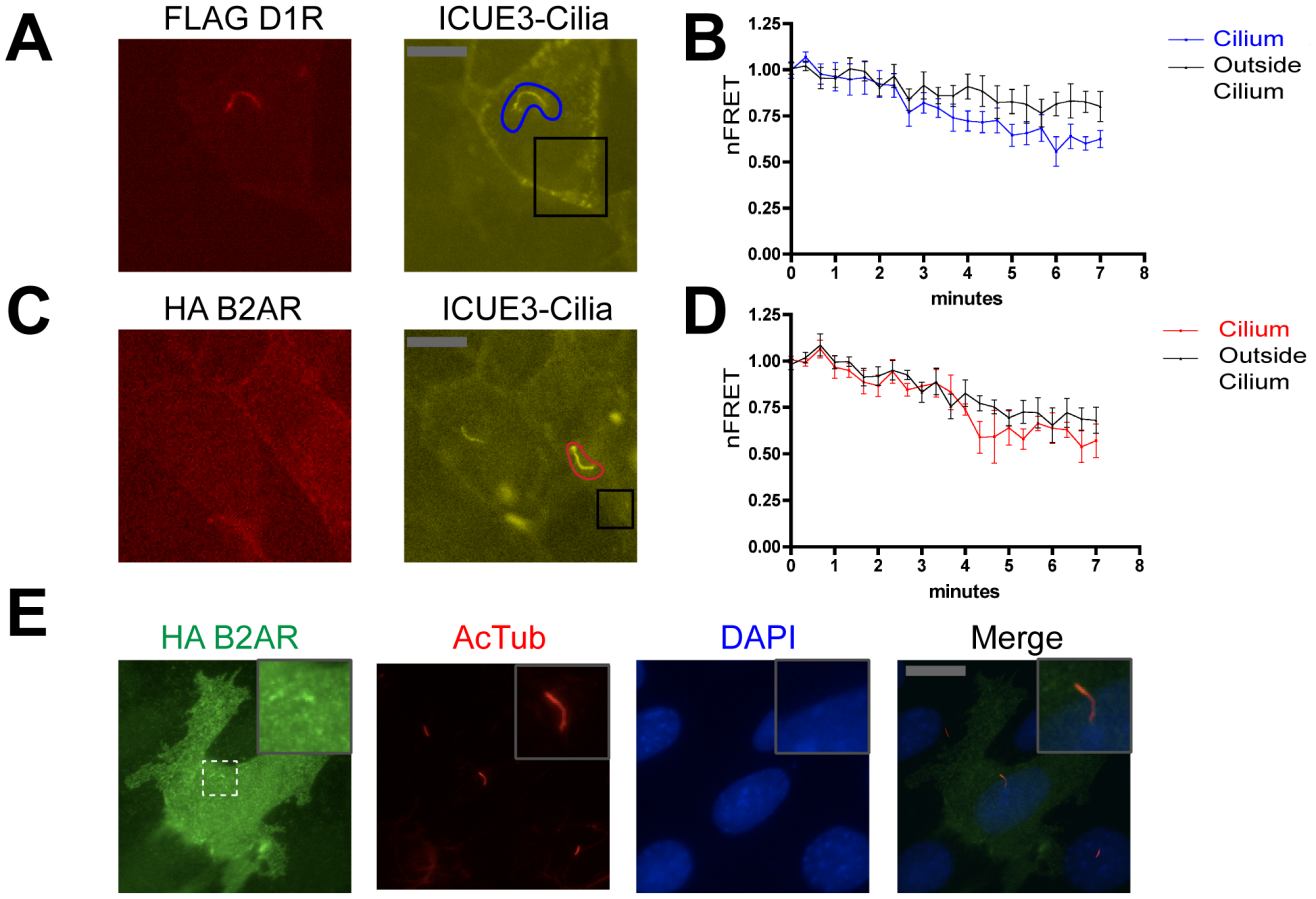
D1R activation drives cAMP accumulation in both ciliary and extra-ciliary domains. **A**. Representative fluorescence micrograph of an IMCD3 cell co-expressing FD1R (left panel, red) and ICUE3-Cilia (right panel, yellow), showing that each localize to the cilium. Blue and black outlines indicate representative examples of ciliary (‘cilium’) and extra-ciliary (‘outside cilium’) ROIs, respectively, used for nFRET determination. Scale bar, 10 um. **B**. Time course of nFRET change in the indicated regions of IMCD3 cells expressing ICUE3-Cilia and Flag-D1R with 1 uM SKF81297 added at the second frame (40 sec). Black line indicates nFRET calculated from the extra-ciliary ROI (‘outside cilum’) and blue line indicates ciliary nFRET (‘cilium’). Results shown are from 8 experiments (≥2 cells imaged per experiment, error bars indicate S.E.M. across experiments). **C**. Representative dual channel fluorescence micrographs showing that HA-B2AR is not enriched in cilia (left panel, red) and ICUE3-Cilia is distributed both inside and outside of cilia (right panel). Red and black outlines indicate representative examples of ciliary (‘cilium’) and extra-ciliary (‘outside cilium’) ROIs, respectively, used for nFRET determination. Scale bar, 10 um. **D**. Time course of nFRET change in the indicated regions of IMCD3 cells co-expressing ICUE3-Cilia and HA-B2AR with 10 uM isoproterenol added at the second frame (40 sec). Black line indicates nFRET calculated from the extra-ciliary ROI (‘outside cilum’) and red line indicates ciliary nFRET (‘cilium’). Results shown are from 8 experiments (≥2 cells imaged per experiment, error bars indicate S.E.M. across experiments). **E**. Representative fluorescence micrograph of HA-B2AR (left panel, green), acetylated tubulin (second panel from left, red), DAPI (third panel from left, blue) and merge (right panel), showing that B2AR is not concentrated in cilia. Scale bar, 10 um. Inset is displayed at 3× increased magnification.

We then used ICUE3-Cilia to examine the location of cAMP accumulation elicited by D1R activation. We first verified that ICUE3-Cilia can colocalize with D1Rs in co-transfected cells (**Fig. 3A**). In these cells, application of the D1R agonist SKF81297 (1 uM) produced a rapid and pronounced decrease in nFRET measured in cilia (**Fig. 3B**, blue line). We also observed a reduced nFRET signal that was nearly as pronounced in magnitude, and occurred with similar kinetics, in the peripheral cytoplasm outside of cilia (**Fig. 3B**, black line). We then carried out the same experiment using a Flag-tagged version of the human B2AR that, in contrast to the D1R, localized diffusely outside of cilia but was not observed in cilia containing co-expressed ICUE3-Cilia (**Fig. 3C**). Acute activation of B2ARs with the selective catecholamine agonist isoproterenol (10 uM) produced a robust decrease in nFRET in the extra-ciliary plasma membrane as expected (Fig. 3D, black line), but we also saw a similar nFRET response within cilia (**Fig. 3D**, red line). Dual imaging of B2AR relative to acetylated tubulin immunoreactivity (and in the absence of ICUE3-Cilia co-expression) further verified that B2ARs are effectively excluded from cilia in IMCD3 cells (**Fig. 3E**). Thus, both cilia-concentrated (D1R) and cilia-excluded (B2ARs) GPCRs can stimulate cAMP accumulation both within and outside of cilia, consistent with rapid diffusion of small cytoplasmic molecules including cAMP into and out of cilia [1], [19].

### GPR88 reveals a discrete function of primary cilia in restricting receptor cross-regulation

**Figure 4:**
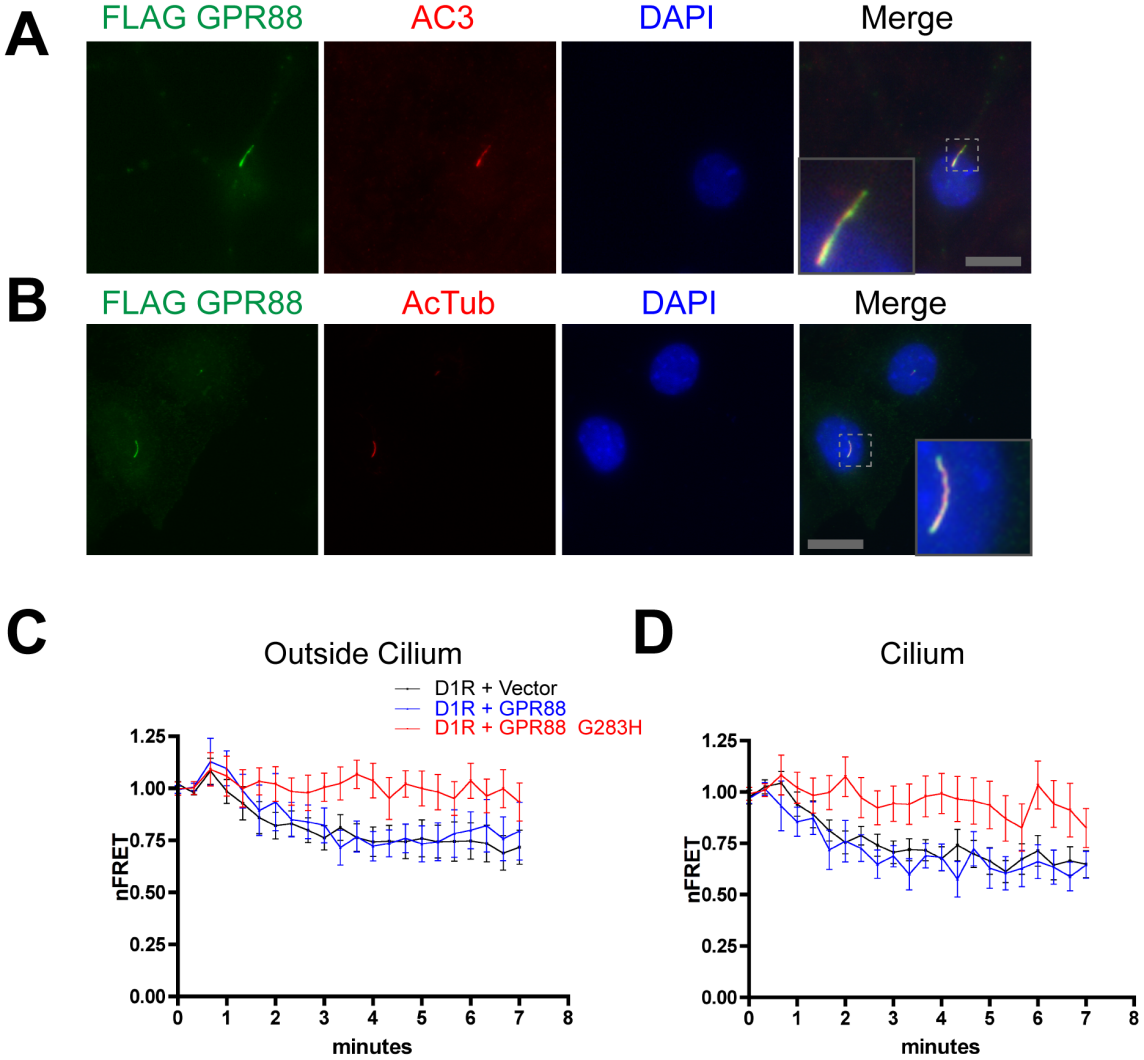
GPR88 localizes to cilia and mutationally activated GPR88 inhibits D1R-dependent cAMP accumulation. **A**. Representative fluorescence micrograph of a cultured striatal neuron showing Flag-GPR88 (green, left panel), the primary cilium marker adenylyl cyclase III, (‘AC3’ red, second panel from left), nuclei are marked by DAPI staining (blue, third panel from left) and merge image showing ciliary GPR88 localization (right panel). Inset shows a higher magnification view of a primary cilium labeled with GPR88. Scale bar, 10 um. Inset is displayed at 3× increased magnification. **B**. Representative fluorescence micrograph showing Flag-GPR88 localization to cilia also in IMCD3 cells. Flag-GPR88 is displayed in green, the ciliary marker acetylated tubulin in red, DAPI in blue, and merge image is at right. Inset shows a representative example at higher magnification. Scale bar, 10 um. Inset is displayed at 4.5× increased magnification. **C**. Time course of ICUE3-Cilia nFRET change outside of cilium measured in IMCD3 cells co-expressing the sensor and Flag-D1R together with vector control (‘D1R+Vector”, black line), HA-tagged wild type GPR88 (‘D1R+GPR88’, blue line) or HA-tagged activated (G283H) GPR88 allele (‘D1R+GPR88_G283H’, red line), and with 1 uM SKF81297 added at the second frame (40 sec). Results shown are from 8 experiments (≥2 cells imaged per experiment, error bars indicate S.E.M. across experiments). **D**. Time course of ICUE3-Cilia nFRET change inside the cilium, determined from the same sets of image series as used to generate the data in panel C, and using the same color scheme for data display. Mutant GPR88 inhibited D1R-mediated cAMP accumulation estimated by nFRET change in both ciliary and extra-ciliary compartments. Results shown are from 8 experiments (≥2 cells imaged per experiment, error bars indicate S.E.M. across experiments).

In the process of screening other brain-expressed GPCRs, we became interested in the orphan GPCR GPR88 because it is present at high levels in the striatum, is co-expressed in striatal medium spiny neurons with D1Rs, and produces large effects on dopaminergic modulation of striatum-dependent behaviors in vivo [10], [11], [20], [21], [22]. An epitope-tagged version of GPR88 prominently concentrated on primary cilia of cultured striatal neurons (**Fig. 4A**) as well as IMCD3 cells (**Fig. 4B**), and did so to a similar degree as the D1R. Accordingly, we wondered if GPR88 might have some effect on D1R-mediated signaling.

To investigate this possibility we used ICUE3-Cilia to monitor the D1R-mediated cAMP response in IMCD3 cells, as above, and examined the effect of co-expressing differentially tagged versions of GPR88. Wild type GPR88 had no detectable effect on the D1R-mediated cAMP response observed in either ciliary or extra-ciliary cytoplasmic compartments (**Fig. 4C and D**, respectively, black and blue curves). However, a point mutation of GPR88 that increases GPR88 activity [23] strongly inhibited cAMP accumulation elicited by the D1R agonist, essentially blocking a detectable response in both cytoplasmic compartments (**Fig. 4C and D**, red curves).

**Figure 5:**
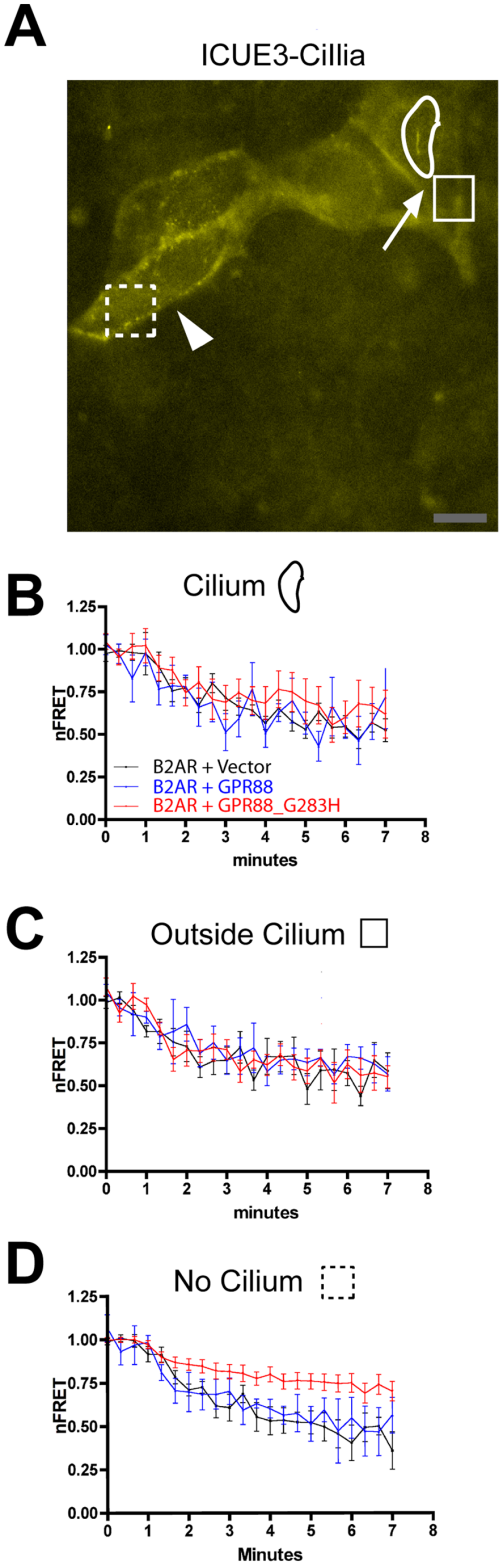
Ciliary localization prevents GPR88 from affecting B2AR signaling. **A**. Representative fluorescence micrograph of co-transfected IMCD3 cells showing ICUE3-Cilia distribution and the corresponding ROIs used for nFRET analysis. A ciliated cell is indicated by white arrow and a non-ciliated cell in the same field is indicated by white arrowhead. Representative examples of ROIs used for determining nFRET are indicated by white outlines. In ciliated cells we resolved ciliary (‘cilium’) and extra-ciliary (‘outside cilium’) compartments; in non-ciliated cells nFRET determination was carried out on a single ROI (‘no cilium’). The example shapes from panel A are copied in the headings to panels B–D, below, in order to further clarify ROIs used for analysis. Scale bar, 10 um. **B**. Time course of the intraciliary ICUE3-Cilia nFRET change determined in ciliated cells (‘cilium’ ROI) co-expressing HA-B2AR and ICUE3-Cilia together with vector control (‘B2AR+Vector”, black line), Flag-tagged wild type GPR88 (‘B2AR+GPR88’, blue line) or Flag-tagged activated (G283H) GPR88 allele (‘B2AR+GPR88_G283H’, red line), and with 10 uM isoproterenol added at the second frame (40 sec). **C**. Time course of the extraciliary ICUE3-Cilia nFRET change in ciliated cells (‘outside cilium’ ROI), displayed using the same color scheme as in Fig. 5B. **D**. Time course of ICUE3-Cilia nFRET change in non-ciliated cells analyzed in the same cultures (‘no cilium’ ROI), displayed using the same color scheme as in Fig. 5B. Mutant GPR88 did not affect B2AR-mediated cAMP accumulation in either cytoplasmic compartment of ciliated cells but strongly inhibited B2AR-mediated cAMP accumulation in non-ciliated cells. Results shown are from 8 experiments (≥2 cells imaged per experiment, error bars indicate S.E.M. across experiments).

To ask if this effect is specific to the D1R, we carried out the same experiments using the B2AR that similarly stimulates overall cytoplasmic cAMP accumulation. Co-expression of wild type GPR88 had no effect on the B2AR-mediated cAMP response measured either inside or outside of cilia (**Fig. 5B and C**, compare black and blue lines), as expected. Interestingly, mutationally activated GPR88, despite its ability to effectively block the D1R-mediated response, did not detectably affect B2AR-mediated cAMP accumulation detected either within or outside of cilia of ciliated cells (**Fig. 5 B and C**, red lines). Thus the B2AR-mediated cAMP response was selectively insensitive to GPR88, relative to the D1R-mediated response that was blocked.

To ask if this pronounced selectivity in D1R relative to B2AR -mediated signal inhibition by GPR88 is a consequence of primary cilia, we took advantage of the fact that ∼40% of IMCD3 cells in our culture preparations lacked cilia, allowing direct comparison of the cAMP response between ciliated and non-ciliated cells imaged in the same microscopic field (**Fig. 5A** shows a representative field, with a ciliated cell indicated by arrow and an adjacent non-ciliated cell indicated by arrowhead). Remarkably, GPR88 strongly inhibited the B2AR-mediated cAMP accumulation but did so only in non-ciliated cells, and in a specific manner requiring mutational activation of GPR88 (**Fig. 5D**). These data suggest that the selective resistance of B2AR-mediated cAMP accumulation to inhibition by GPR88 is indeed a consequence of primary cilia.

## Discussion

The present study investigated the functional significance of primary cilia to conventional GPCR-mediated signaling. We focused on two examples that are activated by diffusible catecholamine ligands, and signal via the diffusible second messenger cAMP, in a simplified ciliated cell culture model. Our results verified ciliary targeting of the D1R, as observed previously in several cell types, and established ciliary exclusion of the B2AR. Primary cilia were not required to facilitate a graded overall cAMP response, and we did not observe a major effect of cilia on localizing the cAMP signal. Instead we found, through investigating the orphan GPCR GPR88, which is targeted to cilia in our model system to a similar degree as the D1R, a discrete effect of primary cilia on the selectivity of GPR88-dependent regulation of the D1R relative to B2AR -mediated signaling response. Because the D1R and B2AR mediate cellular responses to distinct endogenous catecholamines and produce differential effects on target tissues [24], this suggests that primary cilia play a fundamental role in enhancing the selectivity of integrated catecholamine signaling.

The simplest model that is consistent with the present data is that primary cilia function as a compositionally refined plasma membrane microdomain that selectively ‘insulates’ GPR88 from affecting signaling by the cilia-excluded B2AR, while allowing or enhancing GPR88-mediated inhibition of D1R signaling. The biochemical mechanism by which GPR88 mediates this cross-regulation remains to be determined. A simple possibility is that it occurs by reciprocal regulation of local adenylyl cyclase, consistent with the well established ability of Gi to functionally antagonize adenylyl cyclase activation by stimulatory G proteins [25] and evidence that GPR88 couples to Gi [23]. However, this remains to be explicitly tested, and there are other possibilities [26]. The precise biochemical mechanism of GPR88-mediated cross-regulation notwithstanding, the present results identify a discrete function of primary cilia in enhancing signaling selectivity by functionally isolating some GPCRs from others, and doing so in a highly specific manner. Because a limited subset of GPCRs localize to cilia, and otherwise similar receptors can differ in localization relative to cilia, we propose that this insulating function of primary cilia may have more widespread significance.

While our data show that GPR88 is efficiently targeted to primarily cilia in striatal neurons, all of the present functional studies were carried out in IMCD3 cells. These cells provide a simplified model system that is advantageous for experimental manipulation and does not endogenously express D1Rs. Thus it remains to be determined whether the principles described in the present study apply in native cell types. In particular, it will also be important in future work to rigorously determine the actual location of cAMP generation relative to its accumulation, and more fully investigate the degree to which ciliary and extra-ciliary cAMP pools exchange. It is hoped that the ability of ICUE3-Cilia to resolve cAMP dynamics at the level of individual cilia, together with technical improvements in temporal resolution of the analysis, will facilitate future investigation of these important questions.

The presently described function of primary cilia and GPR88 in the regulation of D1R signaling, if it indeed occurs in native neurons, could have significant implications for physiology and disease. GPR88 is co-expressed with D1R in striatum, and GPR88 knockout mice exhibit alterations of striatum-dependent locomotor behaviors and changes in neuronal excitability that are sensitive to D1-specific antagonists [11], [22]. Accordingly primary cilia, and the presently described insulating function of primary cilia, could provide a simple physical basis for GPR88 control of D1R-dependent signaling in vivo. Moreover, because a number of genes linked to major neuropsychiatric disorders influence cilia structure or function [27], we speculate that complex brain diseases might involve compromise of the presently described signal-insulating function of primary cilia.

## Materials and Methods

### cDNA construct and Reagents

Flag-tagged D1 dopamine receptor (human DRD1), Flag-tagged and HA-tagged human beta-2 adrenergic receptor (ADRB2) constructs, cloned into pcDNA3, were described previously [28], [29], [30], [31].

The cAMP biosensor ICUE2 [15], cloned in pcDNA3 and obtained as a generous gift from J. Zhang (Johns Hopkins University), was modified by replacing the citrine present in the biosensor with circularly permuted version of Venus corresponding to the cp194 construct described previously [18] using Sac1 and Not1 flanking sites. A BsiWI site was engineered just 3′ to the initiation codon of this biosensor using site-directed mutagenesis (QuickChange, Agilent). This construct is essentially identical to the previously described ICUE3 [18] except for the addition of an internal BsiWI site, so is hereafter called modified ICUE3. A cDNA encoding the human somatostatin 3 receptor (SSTR3) was obtained from cDNA.org. Site-directed mutagenesis was used to change Asp123 to Glutamate (D123E) in order to disrupt ligand binding to the receptor [16], to add an NheI site just 5′ to the start codon, and to add a BsiWI site just 5′ to the stop codon in frame with the BsiWI site present in modified ICUE3. ICUE3-Cilia was generated by 3 fragment ligation of pIRESneo3 digested with NheI/NotI, modified SSTR3 digested with NheI/BsiWI, and modified ICUE3 digested with BsiWI/NotI. A cDNA encoding human GPR88 was obtained from cDNA.org and modified using PCR to add a signal sequence followed by Flag epitope tag at the N-terminus. A mutant version with Gly283 changed to His (G283H) was constructed to increase basal receptor activity as described [23]. Wild type and mutationally activated versions were cloned into pIRESneo3 or pHUGW [32], [33], respectively.

### Cell culture and transfection

IMCD3 cells (mIMCD-3 cells, ATCC CRL-2123) were cultured in DMEM F-12 supplemented with 10% FBS (UCSF Cell Culture Facility). Cells were transfected with Lipofectamine 2000 (Invitrogen) according to the manufacturer's instructions, with a medium change within 4–6 hours after transfection. Cells were transfected in 6 well dishes and split the day after transfection into 35 mm glass bottom dishes (MatTek, number 1.5).

Dissociated striatal neurons were cultured from embryonic day 17–18 Sprague Dawley rat embryos. All animal procedures were approved by the UCSF Institutional Animal Care and Use Committee (IACUC), and neurons were cultured as described previously [34]. Cells were transfected prior to plating by electroporation (Rat nucleofector kit, Lonza) according to the manufacturer's instructions, plated in 35 mm glass bottom dishes coated with poly-L-lysine (Sigma), changed to Neurobasal media (Invitrogen) supplemented with B27 (Gibco) and L-glutamine (Gibco) 24 h after plating, and experiments were carried out 7 days later.

### Immunocytochemical methods

Surface receptor immunoreactivity was assayed by incubating intact, non-permeabilized IMCD3 cells with rabbit anti FLAG antibody (Sigma, 1 µg/ml for 15 min), washed, and fixed with 3.7% formaldehyde. Surface receptor immunoreactivity for non-permeabilized striatal primary dissociated neurons was assayed by incubating with mouse M1 anti-FLAG antibody (Sigma, 1 µg/ml for 15 min). Cells were washed and permeabilized with 0.1% Triton X- 100 in PBS, 3% milk, then incubated mouse anti acetylated tubulin (Sigma, 1 µg/ml for 60 min) or Rabbit anti AC III (Santa Cruz Biotech, 0.8 µg/ml for 60 min) followed by goat anti-rabbit Alexa594 and goat anti-mouse Alexa488 conjugate (Invitrogen) respectively.

### RNA interference and biochemical determination of cAMP

siRNA duplex targeting IFT88 was obtained from Qiagen (mM_IFT88_4). The sequence of this duplex is r(UUGGAGCUUAUUACAUUGAUA)dTdT and its knockdown efficiency was previously validated (PMID: 20531939). The scrambled control sequence used was r(AAUUCUCCGAACGUGUCACG)dT. Duplexes were transfected using Lipofectamine RNAi-max (Invitrogen) using the optimized protocol provided by the manufacturer for NIH3T3 cells. In all experiments reagent amounts were scaled according to surface area of the specific culture dishes used, based on the optimized protocol listed for 6 well plates. Experiments were conducted 3 days after siRNA transfection without starvation.

To assay cAMP accumulation biochemically, cells expressing FD1 were seeded onto poly-L-lysine-coated 48-well cultures dishes in DMEM/F12HAMs supplemented with 10% fetal bovine serum. For determination of D1R receptor-mediated signaling, the medium was replaced with 0.6 ml of serum-free media containing 0.3 mM 3-isobutyl-1-methylxanthine (IBMX) (Sigma) and the indicated concentration of SKF81297 (Tocris). The cells were then incubated for 15 min at 37°C, and the reaction was terminated by addition of 0.5 ml of 0.1 N HCl/0.1% Triton X-100. cAMP was determined in clarified extracts using a competition enzyme-linked immunosorbent assay obtained commercially (Assay Designs).

### Statistical Analysis

Quantitative data were averaged across multiple independent experiments, with the number of experiments specified in the corresponding figure legend. Unless indicated otherwise, the error bars represent the S.E.M. calculated across experiments calculated using Prism 4.0 software (GraphPad Software, Inc.).

### Determination of cAMP changes by FRET imaging

Cells were imaged using an epifluorescence microscope (Nikon Ti-E) in an enclosed chamber (In Vivo Scientific) at 37°C in a 5% CO2 atmosphere. FRET imaging was carried out essentially as described previously [35], using a Nikon Plan Apo 60×/1.2 WI objective and centering the focal plan on the midpoint of the cilium using a reflection-based autofocus system (Nikon PerfectFocus), capturing the entire cilium and surrounding extra-ciliary plasma membrane within the same focal plane. Cells were imaged in 35 mm glass-bottom dishes (MatTek, no. 1.5) in 2 ml phenol red-free culture medium. Agonists were added by bath application as indicated, using Forskolin (5 nM, Sigma, from 5 uM stock in DMSO) to activate adenylyl cyclase and SKF81297 (1 nM, Sigma, from 1 uM stock in water) to activate D1-type dopamine receptors. Bleaching controls were conducted in each experiment using identical imaging conditions except without agonist or vehicle addition. In some experiments Flag or HA -tagged receptors were simultaneously localized using M1 conjugated to Alexa647 (Sigma) or HA conjugated to Alexa594, (Invitrogen) respectively.

Image analysis was carried out utilizing Nikon Elements AR 4.1 software. ROIs were drawn for extraciliary compartments as indicated in figures (2C, 3A, 5A). ROIs for cilia domains (as indicated in figures 2C, 3A, 5A), were drawn following a maximum projection in order to include the entire cilium throughout the image series (i.e., to allow for ciliary movements occurring over the image series). In each frame, the image present in the ROI was thresholded to select the cilium relative to surrounding extra-ciliary membrane in order to mitigate the effects of cilia movement between time points and between sequential channel exposures of the same time point. To do so, identical thresholded areas for the combined (CFP, FRET, YFP) channel intensity were selected and imposed in register on each channel at each time point within the series (an example is shown in Fig. S1). Background ROIs of similar size were selected in an adjacent cell not expressing biosensor. CFP, FRET, and YFP mean intensities were determined for the indicated ROIs, and exported to Excel for calculation of normalized FRET (nFRET) as also described in [35]. Figures were generated in Prism 4.0 software, with each plot representing averaged results from a minimum of 8 independent experiments per treatment.

## Supporting Information

Figure S1 **Detail of method used to select region of interest used in ciliary FRET calculation.** A three step process was used to define a minimal ROI for ciliary FRET calculation. First, a maximum projection image of the entire time series was generated in NIS Elements using the three channel image displayed in ‘All’ mode. An ROI was manually drawn in this maximum projection to include the entire range of ciliary movement that occurred during the imaging period (panel A, region outlined in red). Second, this aggregate ROI was copied onto each individual image representing a single time point in the series (panel B). Third, within each individual time point ROI, thresholding was used to generate a precise ROI of the ciliary position within that individual image using the ‘Define Threshold’ function in ‘Intensity’ mode of NIS Elements (panel C, purple region). Mean fluorescence intensities, determined within this more restricted ROI for each channel and at each time point, were background-subtracted based on the same ROI applied to an area outside of the cell imaged, and exported to Microsoft Excel for calculation of ciliary nFRET as described in *Materials and Methods*.
(TIF)
