# Supplementary figures and images for: GPR88 Reveals a Discrete Function of Primary Cilia as Selective Insulators of GPCR Cross-Talk
Source: PLoS One. 2013 Aug 2;8(8):e70857. doi: 10.1371/journal.pone.0070857 (PMC3732291; doi:10.1371/journal.pone.0070857)

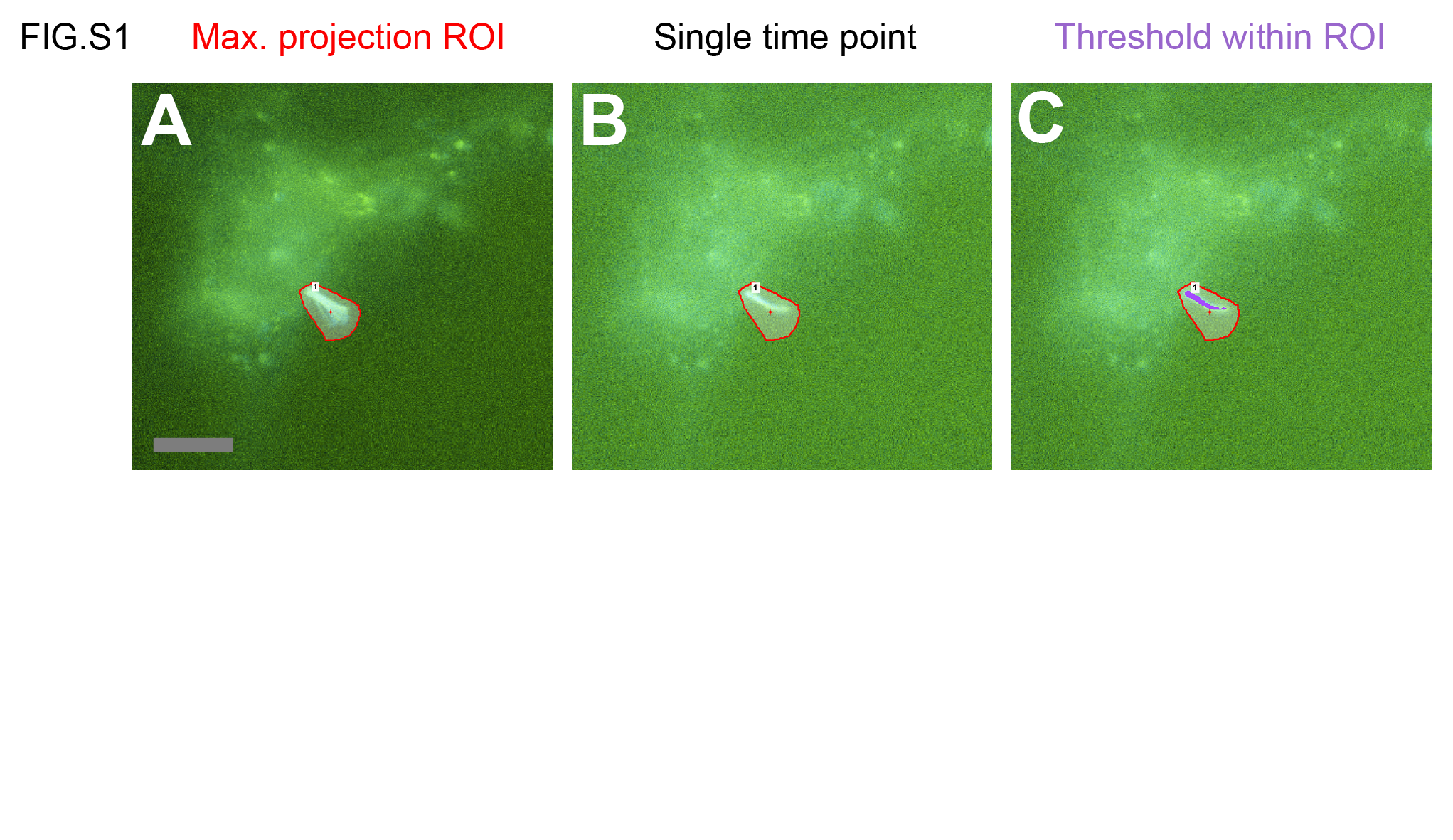

Supplement: Figure S1 — Detail of method used to select region of interest used in ciliary FRET calculation. A three step process was used to define a minimal ROI for ciliary FRET calculation. First, a maximum projection image of the entire time series was generated in NIS Elements using the three channel image displayed in ‘All’ mode. An ROI was manually drawn in this maximum projection to include the entire range of ciliary movement that occurred during the imaging period (panel A, region outlined in red). Second, this aggregate ROI was copied onto each individual image representing a single time point in the series (panel B). Third, within each individual time point ROI, thresholding was used to generate a precise ROI of the ciliary position within that individual image using the ‘Define Threshold’ function in ‘Intensity’ mode of NIS Elements (panel C, purple region). Mean fluorescence intensities, determined within this more restricted ROI for each channel and at each time point, were background-subtracted based on the same ROI applied to an area outside of the cell imaged, and exported to Microsoft Excel for calculation of ciliary nFRET as described in Materials and Methods . (TIF) [file pone.0070857.s001.tif]
